# Comparison of Spectral and Perfusion Computed Tomography Imaging in the Differential Diagnosis of Peripheral Lung Cancer and Focal Organizing Pneumonia

**DOI:** 10.3389/fonc.2021.690254

**Published:** 2021-10-27

**Authors:** Liangna Deng, Guojin Zhang, Xiaoqiang Lin, Tao Han, Bin Zhang, Mengyuan Jing, Junlin Zhou

**Affiliations:** ^1^ Second Clinical School, Lanzhou University, Lanzhou, China; ^2^ Key Laboratory of Medical Imaging of Gansu Province, Lanzhou, China; ^3^ Department of Radiology, Lanzhou University Second Hospital, Lanzhou, China; ^4^ Department of Radiology, Sichuan Provincial People’s Hospital, University of Electronic Science and Technology of China, Chengdu, China

**Keywords:** spectral CT imaging, perfusion CT imaging, peripheral lung cancer, focal organizing pneumonia, differential diagnosis

## Abstract

**Objective:**

To investigate the spectral and perfusion computed tomography (CT) findings of peripheral lung cancer (PLC) and focal organizing pneumonia (FOP) and to compare the accuracy of spectral and perfusion CT imaging in distinguishing PLC from FOP.

**Materials and Methods:**

Patients who were suspected of having lung tumor and underwent “one-stop” chest spectral and perfusion CT, with their diagnosis confirmed pathologically, were prospectively enrolled from September 2020 to March 2021. Patients who were suspected of having lung tumor and underwent “one-stop” chest spectral and perfusion CT, with their diagnosis confirmed pathologically, were prospectively enrolled from September 2020 to March 2021. A total of 57 and 35 patients with PLC and FOP were included, respectively. Spectral parameters (CT_40keV_, CT_70keV_, CT_100keV_, iodine concentration [IC], water concentration [WC], and effective atomic number [Zeff]) of the lesions in the arterial and venous phases were measured in both groups. The slope of the spectral curve (K_70keV_) was calculated. The perfusion parameters, including blood volume (BV), blood flow (BF), mean transit time (MTT), and permeability surface (PS), were measured simultaneously in both groups. The differences in the spectral and perfusion parameters between the groups were examined. Receiver operating characteristic (ROC) curves were generated to calculate and compare the area under the curve (AUC), sensitivity, specificity, and accuracy of both sets of parameters in both groups.

**Results:**

The patients’ demographic and clinical characteristics were similar in both groups (P > 0.05). In the arterial and venous phases, the values of spectral parameters (CT_40keV_, CT_70keV_, spectral curve K_70keV_, IC, and Zeff) were greater in the FOP group than in the PLC group (P < 0.05). In contrast, the values of the perfusion parameters (BV, BF, MTT, and PS) were smaller in the FOP group than in the PLC group (P < 0.05). The AUC of the combination of the spectral parameters was larger than that of the perfusion parameters. For the former imaging method, the AUC, sensitivity, and specificity were 0.89 (95% confidence interval [CI]: 0.82–0.96), 0.86, and 0.83, respectively. For the latter imaging method, the AUC, sensitivity, and specificity were 0.80 (95% CI: 0.70–0.90), 0.71, and 0.83, respectively. There was no significant difference in AUC between the two imaging methods (P > 0.05).

**Conclusion:**

Spectral and perfusion CT both has the capability to differentiate PLC and FOP. However, compared to perfusion CT imaging, spectral CT imaging has higher diagnostic efficiency in distinguishing them.

## Introduction

Focal organizing pneumonia (FOP) is a subtype of organizing pneumonia, accounting for approximately 10–15% of organizing pneumonia cases (1). It often presents as a solitary nodule or mass on chest computed tomography (CT) and is associated with atypical symptoms and imaging findings that overlap with those of peripheral lung cancer (PLC) (2). In fact, FOP is often misdiagnosed as PLC and overtreated with procedures such as surgical resection and puncture biopsy, which increase the distress levels of patients, the likelihood of complaints, and the overall cost of treatment (3). In addition, needle biopsy may cause the risk of pneumothorax, bleeding, and even tumor metastasis, and the small tissue biopsy sample obtained for organizing pneumonia is often insufficient to rule out malignant lung tumors, which often coexist with inflammation (3, 4). Therefore, a method that allows to non-invasively distinguish PLC from FOP before surgery is required.

CT is the preferred imaging method for lung lesions. However, traditional CT can only provide information about the morphology of the lesion and its relationship with adjacent tissues. 18F-deoxyglucose positron emission tomography can provide more information about the functional metabolism of the lesion, but its use in clinical practice is limited due to its high cost and excessive radiation dose (5, 6). In recent years, with the rapid development of CT functional imaging, spectral and perfusion CT imaging have shown advantages in the diagnosis and differentiation of lung tumors (7–9). Spectral CT imaging can provide a variety of imaging parameters, such as single-energy image, spectral curve, effective atomic number (Zeff), and basic material image, which has potential value in the qualitative analysis of lesions and determination of tumor grade and origin (10, 11). Perfusion CT achieves a relatively non-invasive dynamic observation of the tissue blood perfusion state from the capillary level in the living body and more intuitively reflects the rich blood supply, blood supply characteristics, and hemodynamic changes of the lesion (12, 13). Yu et al. (14) evaluated the use of spectral CT to image malignant tumors and inflammatory masses; they found that the area under the curve (AUC) of the CT value, slope of spectral curve, and normalized iodine concentration (IC) values were all greater than 80%, and the sensitivity and specificity values of the venous phase with normalized IC were 86% and 100%, respectively. Yuan et al. (15) showed that the perfusion index has the highest diagnostic efficiency in distinguishing benign from malignant pulmonary nodules, with sensitivity and specificity of 95% and 83%, respectively. Taken together, this evidence suggests that spectral or perfusion CT imaging is used to distinguish benign from malignant lung masses. However, no previous study has compared the accuracy of spectral and perfusion CT imaging in the differential diagnosis of benign and malignant lung tumors. In this study, we prospectively enrolled patients who underwent “one-stop” spectral and perfusion CT to compare the diagnostic accuracy of these CT modalities in the differential diagnosis of PLC and FOP.

## Materials and Methods

### General Information

**Figure 1:**
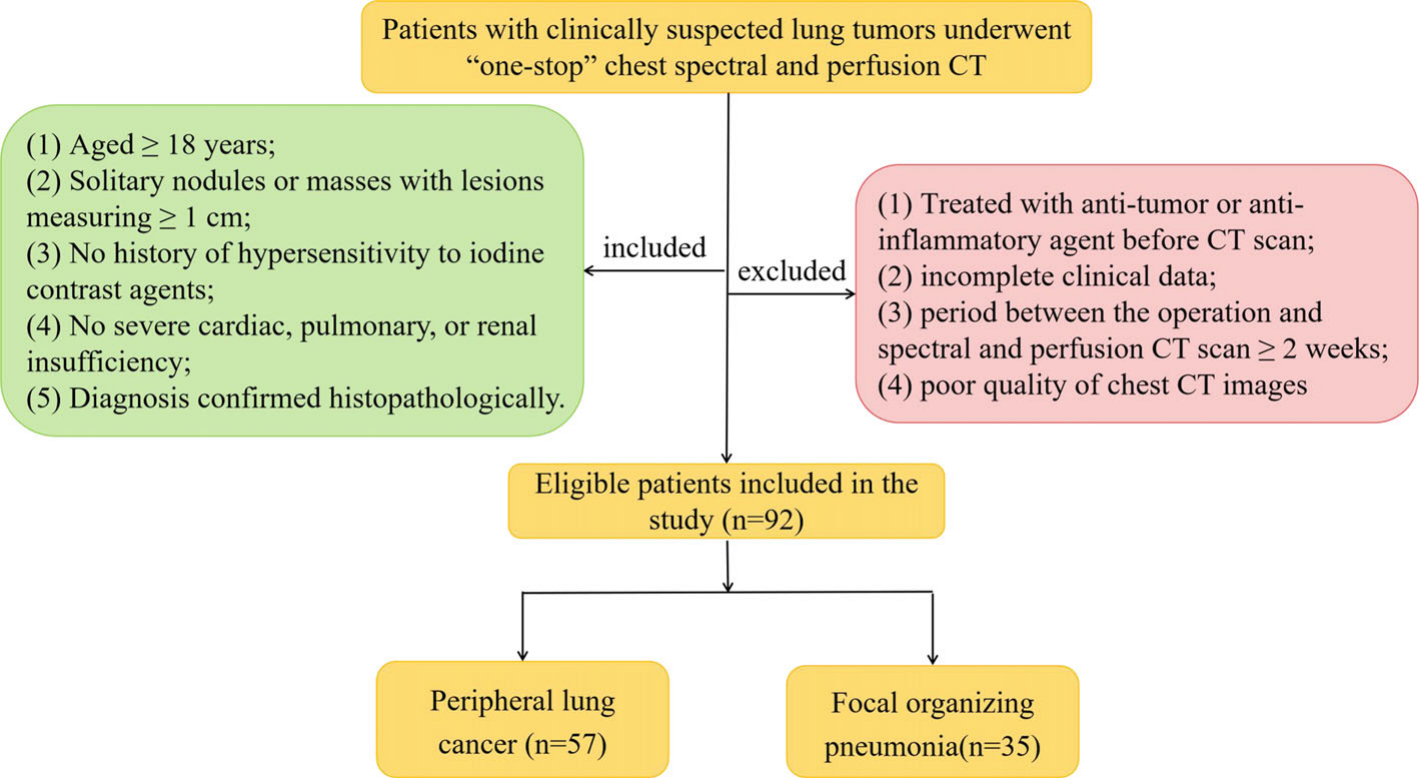
Flowchart of inclusion and exclusion criteria.

We prospectively enrolled 92 patients with clinically suspected lung tumors, presenting at our hospital between September 2020 and March 2021. The patients underwent “one-stop” chest spectral and perfusion CT; their diagnosis was confirmed pathologically. There were 57 patients with PLC (31 males and 26 females, mean [± standard deviation, SD] age, 58.61 ± 10.95 [range, 31–74] years) and 35 patients with FOP (22 males and 13 females, mean [± SD] age, 56.09 ± 12.11 [range, 20–76] years). Patients were eligible for the present study if they met the following inclusion criteria: (1) aged ≥ 18 years and able and willing to provide consent; (2) chest CT showing solitary nodules or masses with lesions measuring ≥ 1 cm; (3) no history of hypersensitivity to iodine contrast agents; (4) no severe cardiac, pulmonary, or renal insufficiency; and (5) diagnosis confirmed histopathologically. The exclusion criteria were as follows: (1) treated with an anti-tumor or anti-inflammatory agent before undergoing CT, (2) incomplete clinical data, (3) period between the operation and spectral and perfusion CT scan ≥ 2 weeks, and (4) poor quality of chest CT images (including respiratory motion artifacts or body surface metal artifact). **Figure 1** presents the flowchart of the inclusion and exclusion criteria. The study was approved by the Institutional Review Board and Ethics Committee of the Lanzhou University Second Hospital (Lanzhou, China). Written informed consent was obtained from all patients.

### Instruments and Methods

All patients underwent imaging using 256-slice Revolution CT scanner (GE Healthcare, Milwaukee, WI, USA). First, the patients underwent a routine chest CT, with the scanning range from the thoracic entrance to the costal diaphragmatic angle level. The location and scope of the lesion were determined according to the plain scan image, and the chest “one-stop” spectral and perfusion CT was performed with the largest level of the lesion as the center. We used a deconvolution algorithm to compute quantitative perfusion CT data, which is based on the convolution model. Perfusion scanning parameters were as follows: tube voltage, 80–100 kVp; tube current, 100 mA; pitch, 0.984:1; tube rotation time, 0.5 s; scanning field of view, 50 cm; collimation width, 128 × 0.625 mm; Asir-V, 40%; and scanning layer thickness and layer spacing, 5 mm. Spectral scanning parameters were as follows: 80 kVp tube voltage and 140 kVp instantaneous switching; the tube current was automatically modulated, and other parameters were the same as those for CT perfusion. A total of 50–80 mL (1.3 mL/kg body weight) non-ionic iodine contrast agent (Ultravist 300, Bayer Pharma, Berlin, Germany) was injected into the anterior cubital vein with a high-pressure syringe (XD8000, Ulrich, Germany) at a flow rate of 4–5.5 mL/s (<50 kg, 4 mL/s; 50–69 kg, 4.5 mL/s; 70–89 kg, 5 mL/s; >90 kg, 5.5 mL/s) (13, 16). Scanning began 2 s after the contrast agent was injected, and images were collected every 2 s (exposure time, 0.5 s; interval, 1.5 s), for a total of 14 times during the inflow period; during the outflow period, images were collected every 3 s (exposure time, 0.5 s; interval, 2.5 s), five times. A total of 21 images were collected during the entire perfusion process. A two-phase spectral enhancement was performed 30.2 s and 52.6 s after the injection of a contrast agent. Moreover, 60% Asir-V iteration was used to reconstruct the two-phase spectral-enhanced image horizontally at the end of the scan; layer thickness and spacing remained at 1.25 mm. The radiation dose parameters of perfusion and spectral CT (arterial and venous phase) scan sequences were systematically collected.

### Computed Tomography Image Analysis

All images were examined by two radiologists with 4 and 8 years of experience diagnosing chest tumors, respectively; both radiologists were blinded to patient data. The perfusion images were corrected using the CT Dynamic Registration software in GE AW4.7 workstation and then analyzed using the CT Perfusion 4D software. The thoracic aorta was selected as the input artery, and the pulmonary artery was used as the output artery to place the region of interest (ROI). The area of the ROI was approximately 1/2–2/3 of the cross-sectional area of the blood vessel. The time-density curve (TDC) of the thoracic aorta-pulmonary artery was generated automatically, and pseudo-color maps of various perfusion parameters were obtained. The ROI was manually outlined on three consecutive levels, including the largest level of the lesion and its adjacent upper and lower levels. The ROI was delineated to avoid calcification, blood vessels, cavities, atelectasis, and necrotic cysts that may affect measurements. Take the average of three measurements for each case. Then the average value obtained from the measurements of the two radiologists was then calculated again as the mean value. the scores from the assessing radiologists were also averaged. The perfusion parameters were as follows: blood volume (BV), blood flow (BF), mean transit time (MTT), and permeability surface (PS). The GSI Viewer software was used to analyze the spectral image with the same ROI measurement method. The CT values at a single-energy level of 40–140 keV (interval of 10 keV), IC, water concentration (WC), and Zeff values were obtained, and the slope of the spectral curve was calculated. The formula used to calculate the slope was K_70 keV_ = (CT_40keV_-CT_70keV_)/ (70–40).

### Statistical Analysis

Statistical analysis was performed using the Statistical Package for the Social Sciences version 23.0 (International Business Machines Corporation, Armonk, NY, USA). Enumerative data are expressed as percentages, and quantitative data are expressed as mean ± SD. The distributions of sex, smoking history, and clinical characteristics were compared between the groups using the *χ* ^2^ test and Fisher’s exact test. Between-group differences in quantitative variables were compared using the two-sample t-test or Mann-Whitney U test. The intraclass correlation coefficient (ICC) was used to evaluate the repeatability of the measurement results of two radiologists. The receiver operating characteristic (ROC) curve was created for the variables that were significantly different between the groups, and the AUC was calculated to evaluate the diagnostic efficacy of the two imaging methods. The DeLong test was used to compare whether the efficiency difference between the two methods was statistically significant.

## Results

A comparison of the clinical features of PLC and FOP is shown in **Table 1**. There was no between-group difference in the distributions of sex, age, smoking history, or symptoms between the PLC and FOP groups (P > 0.05).

**Table 1:** Demographic and clinical features of patients with PLC and FOP.

Variable	PLC(n=57)	FOP(n=35)	*χ* ^2^/*t*	*P*
Sex (%)			0.64	0.43
Male	31(54.4%)	22(62.9%)		
Female	26(45.6%)	13(37.1%)		
Age(years)			-1.03	0.31
Mean ± SD	58.61 ± 10.95	56.09 ± 12.11		
Median/Range	59 (37–81)	56(20-76)		
Smoking history (%)^*^			0.05	0.83
Yes	28(49.1%)	18(51.4%)		
No	29(50.9%)	17(48.6%)		
Symptoms (%)			3.71-*	0.73
Asymptomatic	12(11.2%)	8(20.0%)		
Cough	30(28.0%)	9(22.5%)		
Sputum	27(25.2%)	7(17.5%)		
Dyspnea	11(10.3%)	3(7.5%)		
Hemoptysis	8(7.5%)	4(10%)		
Chest pain	12(11.2%)	5(12.5%)		
Fever	7(6.5%)	4(10%)		

FOP, Focal organizing pneumonia; PLC, Peripheral lung cancer; SD, Standard deviation. *Smoking history is defined as follows: Yes, former and current smokers; No, never smoked. -* Fisher’s exact test.

**Figure 2:**
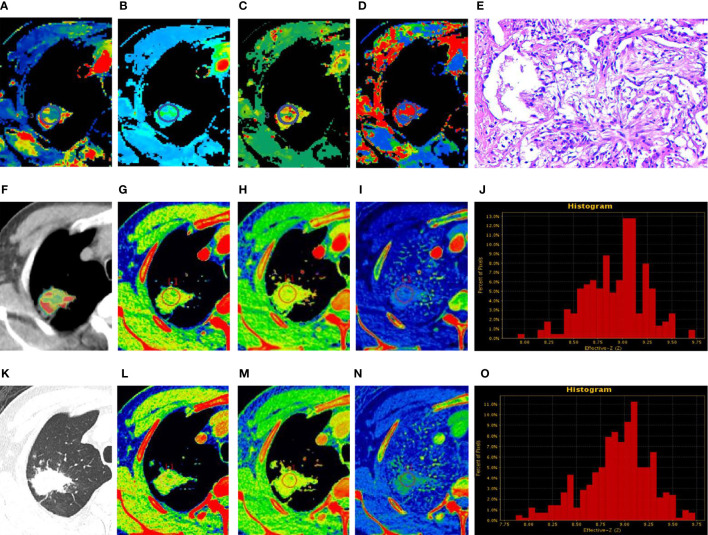
Focal organizing pneumonia. **(A–D, F)** Perfusion computed tomography (CT) fusion pseudo-color map; **(E)** Hematoxylin and eosin staining (original magnification ×200); **(G–J)** Spectral CT images of arterial phase (AP); **(K)** Lung window; **(L–O)** Spectral CT images of venous phase (VP). **(A–D)** Blood flow, blood volume, permeability surface, and average transit time values of the lesions were 63.74 mL/100 g·min, 10.29 mL/100 g, 25.06 mL/100 g·min, 16.09 s, respectively. **(G, L)** 70 keV single-energy pseudocolor image (CT number of 70 keV were 79.27 HU and 75.9 HU, respectively). **(H, M)** The effective atomic numbers (Zeff) pseudocolor image (Zeff were 8.93 and 8.81, respectively). **(I, N)** The iodine-based material decomposition image (iodine concentration were 22.98 100µg/cm^3^ and 21.05 100 µg/cm^3^, respectively). **(J, O)** The histogram of Zeff.

**Figure 3:**
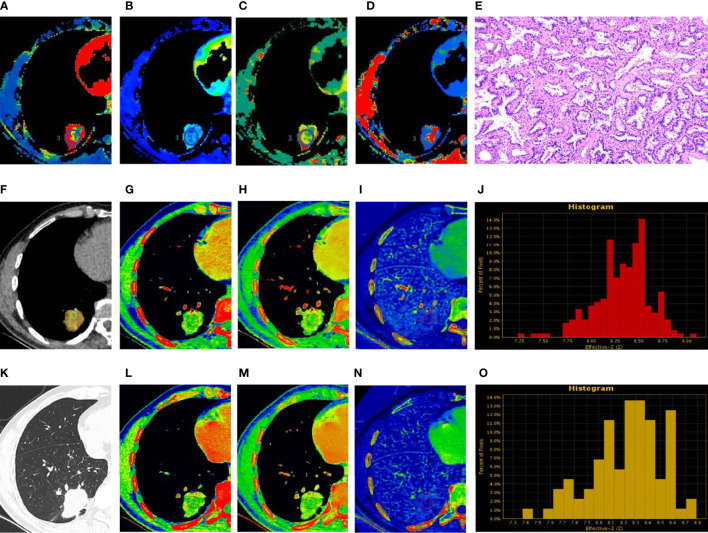
Peripheral lung cancer (PLC). **(A–D, F)** Perfusion computed tomography (CT) fusion pseudo-color map; **(E)** Hematoxylin and eosin staining (original magnification ×100); **(G–J)** Spectral CT images of arterial phase (AP); **(K)** Lung window; **(L–O)** Spectral CT images of venous phase (VP). **(A–D)** Blood flow, blood volume, permeability surface, and average transit time values of the lesions were 73.52 mL/100 g·min, 12.3 mL/100 g, 28.89 mL/100 g·min, 15.57 s, respectively. **(G, L)** 70 keV single-energy pseudocolor image (CT number of 70 keV were 76.03 HU and 71.03 HU, respectively). **(H, M)** The effective atomic numbers (Zeff) pseudocolor image (Zeff were 8.88 and 8.61, respectively). **(I, N)** The iodine-based material decomposition image (iodine concentration were 20.84 100µg/cm^3^ and 16.98 100 µg/cm^3^, respectively). **(J, O)** The histogram of Zeff.

The estimates of spectral parameters and their comparisons between the PLC and FOP groups are presented in **Table 2**. The two radiologists had strong consistency in differentiating PLC from FOP. The ICC values were all > 0.80. In the arterial and venous phases, the values of the spectral parameters (CT_40keV_, CT_70keV_, K70 keV, IC, and Zeff) were greater in the FOP group than in the PLC group, and the difference was statistically significant (P < 0.05). There was no between-group difference in the values of WC or CT _100keV_ (P > 0.05). The estimates of perfusion parameters and their comparisons between the PLC and FOP groups are presented in **Table 3**. The estimates of the perfusion parameters (BV, BF, MTT, and PS) were all greater in the PLC group than in the FOP group (P < 0.05). **Figures 2** and **3** present examples of PLC and FOP, respectively.

**Table 2:** Comparison of spectral quantitative parameters of PCL and FOP in AP and VP.

Parameter	PLC(n=57)	FOP(n=35)	*t*	*P*
AP				
CT_40keV_ (HU)	187.67 ± 17.41	202.51 ± 15.73	-4.11	<0.001
CT_70keV_ (HU)	78.71 ± 7.25	82 ± 5.94	-2.27	0.03
CT_100keV_ (HU)	50.39 ± 6.44	50.68 ± 5.33	-0.22	0.83
K_70keV_	3.63 ± 0.43	4.02 ± 0.40	-4.27	<0.001
IC (100µg/cm^3)^	19.31 ± 2.28	21.35 ± 2.15	-4.27	<0.001
WC (mg/cm3)	1028.50 ± 6.93	1026.49 ± 6.06	1.42	0.16
Zeff	8.73 ± 0.12	8.84 ± 0.11	-4.28	<0.001
VP				
CT_40keV_ (HU)	173.05 ± 19.78	191.55 ± 25.38	-3.68	0.001
CT_70keV_ (HU)	73.89 ± 8.26	78.37 ± 11.08	-2.07	0.04
CT_100keV_ (HU)	48.46 ± 6.76	48.98 ± 7.98	-0.34	0.74
K_70keV_	3.31 ± 0.45	3.77 ± 0.51	-4.62	<0.001
IC (100µg/cm^3)^	17.48 ± 2.36	20.04 ± 2.71	-4.77	<0.001
WC (mg/cm3)	1028.65 ± 6.58	1026.24 ± 6.30	1.73	0.09
Zeff	8.63 ± 0.13	8.77 ± 0.14	-4.68	<0.001

AP, Arterial phase; FOP, Focal organizing pneumonia; HU, Hounsfield; IC, iodine concentration; PLC, Peripheral lung cancer; VP, Venous phase; WC, water concentration; Zeff, effective atomic number.

**Table 3:** Comparison of perfusion parameters of PCL and FOP.

Parameters	PLC (n=57)	FOP (n=35)	*t/Z*	*P*
BV (mL/100 g·min)	7.48 ± 1.48	6.33 ± 1.54	3.54	0.001
BF (mL/100g)	94.66 ± 42.17	81.71 ± 12.38	2.17	0.03
MTT (s)	10.58(8.10-12.01)	5.78(4.68-9.26)	3.89	<0.01
PS (mL/100 g·min)	22.39 ± 2.99	20.58 ± 4.23	2.21	0.03

FOP, Focal organizing pneumonia; PLC, Peripheral lung cancer; BF, Blood flow; BV, Blood volume; MTT, Mean transit time; PS, permeability surface.

**Figure 4:**
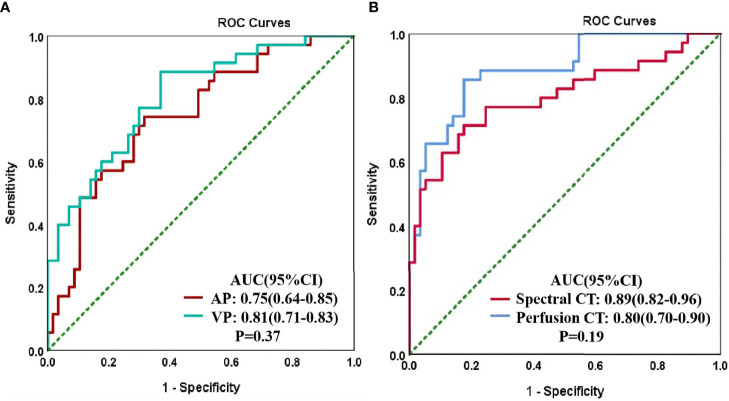
Receiver operating characteristic (ROC) curves for CT spectral parameters in arterial (AP) and venous (VP) phases in the peripheral lung cancer (PLC) and focal organizing pneumonia (FOP) groups **(A)**. The ROC curves of spectral and perfusion imaging for the PLC and FOP groups **(B)**.

The ROC curve findings are presented in **Table 4** and **Figure 4**. The AUC of the spectral parameters was 0.89, and the corresponding sensitivity, specificity, and accuracy estimates were 0.86, 0.83, and 0.84, respectively. Moreover, the AUC of the perfusion parameters was 0.80, and the corresponding sensitivity, specificity, and accuracy estimates were 0.71, 0.83, and 0.78, respectively. The AUC and accuracy estimate of the spectral parameters in the venous phase (0.81, 0.73) were larger than those in the arterial phase (0.75, 0.70). The DeLong test showed that there was no significant difference in AUC between the two imaging methods and the spectral parameters of the two phases (P > 0.05).

**Table 4:** ROC curve analysis of spectral and perfusion parameters.

Parameters	AUC (95%CI)	YI	Sensitivity (%)	Specificity (%)	Accuracy (%)
Spectral parameters					
Ap	0.75 (0.64-0.85)	0.43	0.74	0.68	0.70
VP	0.81 (0.71-0.83)	0.52	0.89	0.63	0.73
Ap + VP	0.89 (0.82-0.96)	0.68	0.86	0.83	0.84
perfusion parameters	0.80 (0.70-0.90)	0.54	0.71	0.83	0.78

AP, Arterial phase; AUC, Area under cure; VP, Venous phase; YI, Youden index.

The dose-length product and volume CT dose index estimates of the perfusion CT were 320.78 ± 1.21 mGy·cm and 20.04 mGy, respectively; the corresponding values for spectral CT were 622.63 ± 51.13 mGy·cm and 15.9 mGy, respectively.

## Discussion

In this study, our findings suggest that both spectral and perfusion CT imaging are associated with diagnostic efficiency at distinguishing PLC from FOP. However, spectral CT imaging has some advantages over perfusion CT imaging.

Our results suggest that spectral imaging is associated with greater diagnostic efficiency than perfusion imaging in distinguishing PLC from FOP, with sensitivity, specificity, and accuracy estimates of 86%, 83%, and 84%, respectively. The diagnostic efficiency of spectral imaging was higher in the venous phase (AUC, 81%) than in the arterial phase (AUC, 75%), and the diagnostic efficacy of the combination of arterial and venous phases was particularly high (AUC, 89%). These findings are consistent with those of a previous study (17). This may be because the contrast agent cannot completely enter the capillaries and intercellular spaces in the arterial phase; thus, the parameters of the venous phase can better reflect the histological characteristics of the lesion (18). Moreover, we found that the spectral parameters of FOP in the arterial and venous phase are greater than those of PLC. This may be due to the pathophysiological basis of the two and the difference between the two imaging methods. FOP is stimulated by inflammatory factors to cause vasodilation and increased microvascular permeability (2). However, the microvascular arrangement formed by PLC is intricate and tortuous, and the contrast agent flows slowly in the tumor tissue (19). However, the perfusion parameters of PLC are greater than those of FOP. Some of the requirements of CT perfusion analysis are to select the blood vessel supplying the lesion, obtain the TDC by placing the ROI on the blood supply vessel, and then compare it with the TDC of the lesion to obtain the lesion characteristics of blood supply (13). The lung is supplied by both pulmonary and bronchial arteries, and there are differences between the main blood supply vessels of PLC and FOP (15). Moreover, with the circulation of blood, the parameters measured at different times will be different, which may cause the inconsistency between the spectral and the perfusion parameters. In addition, perfusion imaging showed good efficacy in differentiating PLC from FOP in the present study (AUC, 80%). This is broadly consistent with the results of previous studies (15, 20). Although the performance of the results of these studies is different, it shows that it is feasible to distinguish benign from malignant lung tumors by CT perfusion. However, these studies only identified benign and malignant pulmonary nodules and did not further classify these nodules. In clinical practice, it is difficult to distinguish PLC from FOP, and FOP is often misdiagnosed as a malignant tumor and thus overtreated. Therefore, the benign lesions in our study only included patients with FOP, these cases are all lesions of the same nature, and the differences between groups will be smaller. In this study, compared with perfusion imaging, spectral imaging showed a higher diagnostic efficiency in distinguishing PLC from FOP, but there was no statistical significance between the two. This may be related to our small sample size, which needs to be further expanded for verification in the future.

With the rapid development of spectral and perfusion CT imaging technology, it is widely used in clinical settings. Spectral CT imaging can obtain single-energy images and base material density values at different keV levels and calculate the Zeff value of the lesion based on the obtained spectral curve. Spectral CT imaging provides various quantitative parameters and tools for lesion analysis, which may support qualitative analysis and treatment assessment of lesions (21, 22). CT perfusion imaging can reflect the structural and functional differences between normal vessels and neovascularization in different tissues (23–25). However, CT perfusion imaging is associated with some limitations. First, it requires the acquisition of multiple consecutive scans of the lesion, which increases patients’ exposure to radiation. Second, CT imaging perfusion parameters are affected by factors such as the kinetic model of perfusion imaging, contrast agent concentration, and contrast injection rate (23, 26, 27). In conclusion, perfusion CT involves a net increase in patient’s radiation exposure, and its findings could be highly variable based on ROI selection and the computational model used to process CT perfusion data, whereas spectral CT would at least not require any additional radiation exposure and could be more robust and easier to use/interpret. In addition, some studies have shown a good correlation between spectral and perfusion parameters in many types of tumors (28–30). Moreover, the growing workload increases radiologists’ stress and may affect diagnostic performance. Therefore, proper imaging examinations for patients can reduce unnecessary imaging examinations and can minimize the waste of medical resources (31). Overall, this evidence suggests that spectral CT is the first-line approach for evaluating tumor hemodynamics in patients with poor respiratory function and in those requiring a low radiation dose.

To the best of our knowledge, this is the first study to use “one-stop” spectral and perfusion scanning and to compare the diagnostic effectiveness of these two imaging methods. Both spectral and perfusion CT imaging types can distinguish benign from malignant tumors (32), especially chest tumors (14, 15, 19, 33, 34). However, previously reported estimates of diagnostic efficacy have been inconsistent, and the conclusions remain subject to debate. In our study, the “one-stop” approach to spectral and perfusion scanning helped obtain multiple quantitative parameters for both imaging types within a single scan; this combined approach yielded comprehensive information on tumor histological characteristics. Further, this “one-stop” approach required a single injection of the contrast agent, allowing to maintain its use to the minimum while reducing patients’ exposure to radiation. We also used Asir-V, which indirectly allows lowering radiation dose by reducing the higher noise derived from lower dose scans (35). Most importantly, through a single scan, the spectral and perfusion data obtained are consistent in space and time, which makes our research results more accurate.

This study has some limitations. First, this was a preliminary study with a small sample size, and the results may thus be overestimated. However, we indicated that our results are aligned with those previously published. Future studies should validate the present findings using larger samples. Second, we only measured these parameters at the largest level of the lesion rather than based on the volume of the entire lesion; thus, the effect of spatial heterogeneity was ignored when measuring the lesion. However, due to current technical limitations, we can only perform measurements on a two-dimensional level. Finally, different kinetic models used in perfusion CT imaging will lead to different perfusion parameters obtained; thus, the results of our study need to be verified by other types of kinetic models.

In conclusion, this prospective study found no significant difference between spectral and perfusion CT in distinguishing PLC from FOP. Both modalities has the capability to differentiate PLC and FOP. However, compared to perfusion CT imaging, spectral CT imaging has higher diagnostic efficiency in distinguishing them.

## Data Availability Statement

The raw data supporting the conclusions of this article will be made available by the authors, without undue reservation.

## Ethics Statement

The studies involving human participants were reviewed and approved by The Ethics Committee of the Second Hospital of Lanzhou University (Lanzhou, China). The patients/participants provided their written informed consent to participate in this study.

## Author Contributions

All authors contributed to the conception or design of the work, the acquisition, analysis, or interpretation of data for the work, and the drafting of the work or revising it critically for important intellectual content. All authors contributed to the article and approved the submitted version. LD collected all changes and to upload the revised files.

## Conflict of Interest

The authors declare that the research was conducted in the absence of any commercial or financial relationships that could be construed as a potential conflict of interest.

## Publisher’s Note

All claims expressed in this article are solely those of the authors and do not necessarily represent those of their affiliated organizations, or those of the publisher, the editors and the reviewers. Any product that may be evaluated in this article, or claim that may be made by its manufacturer, is not guaranteed or endorsed by the publisher.

## References

[1] Shen L Jing L Huang L Zhang Y Hong Y. Cryptogenic Organizing Pneumonia Presenting as a Solitary Mass: Clinical, Imaging, and Pathologic Features. Med Sci Monit (2019) 25:466–74. doi: 10.12659/MSM.911655 PMC6343522 30648699

[2] Zhao F Yan SX Wang GF Wang J Lu PX Chen B. CT Features of Focal Organizing Pneumonia: An Analysis of Consecutive Histopathologically Confirmed 45 Cases. Eur J Radiol (2014) 83(1):73–8. doi: 10.1016/j.ejrad.2013.04.017 23711424

[3] Zhi Z Pan Y Song C Hao W Wu S Xiang W. Focal Organizing Pneumonia Mimicking Lung Cancer: A Surgeon’s View. Am Surg (2012) 78(1):133–7. doi: 10.1177/000313481207800150 22273330

[4] Aranda R. Organizing Pneumonia Adjacent to Lung Cancer: Frequency and Clinico-Pathologic Features. Lung Cancer (2002) 35(2):195–201. doi: 10.1016/s0169-5002(01)00405-6 11804693

[5] Baxa J Vondráková A MatoušKová T Růžičková O Schmidt B Flohr T. Dual-Phase Dual-Energy CT in Patients With Lung Cancer: Assessment of the Additional Value of Iodine Quantification in Lymph Node Therapy Response. Eur Radiol (2014) 24(8):1981–8. doi: 10.1007/s00330-014-3223-9 24895031

[6] Chae EJ Song JW Seo JB Krauss B Song KS. Clinical Utility of Dual-Energy CT in the Evaluation of Solitary Pulmonary Nodules: Initial Experience. Int J Med Radiol (2009) 249(2):671–81. doi: 10.1148/radiol.2492071956 18796658

[7] Jia X Sun J. CT Spectral Parameters and Serum Tumour Markers to Differentiate Histological Types of Cancer Histology. Clin Radiol (2018) 73(12):1033–40. doi: 10.1016/j.crad.2018.07.104 30115364

[8] Zhang G Cao Y Zhang J Zhao Z Zhou J. Epidermal Growth Factor Receptor Mutations in Lung Adenocarcinoma: Associations Between Dual-Energy Spectral CT Measurements and Histologic Results. J Cancer Res Clin Oncol (2020) 147(4):1169–78. doi: 10.1007/s00432-020-03402-8 PMC11802015 32980961

[9] Ovali GY Sakar A GöKtan C Celik P Yorgancioglu A Nese N. Thorax Perfusion CT in Non-Small Cell Lung Cancer. Computerized Med Imaging Graphics (2007) 31(8):686–91. doi: 10.1016/j.compmedimag.2007.08.005 17904334

[10] Matsumoto K Jinzaki M Tanami Y Yamada M Nakajima K eds. Virtual Monochromatic Spectral Imaging With Fast kV Switching: Improvement of Image Quality Compared With Conventional 120kvp CT. In: Radiological Society of North America 2010 Scientific Assembly and Annual Meeting. New York, America.

[11] Lv P Zhang Y Liu J Ji L Chen Y Gao J. Material Decomposition Images Generated From Spectral CT: Detectability of Urinary Calculi and Influencing Factors. Acad Radiol (2014) 21(1):79–85. doi: 10.1016/j.acra.2013.09.023 24331268

[12] Antonietta MM Lorenzo P Alessandro C Luca V. CT Perfusion: Technical Developments and Current and Future Applications. BioMed Res Int (2016) 2015:1–2. doi: 10.1155/2015/397521 PMC4324810 25695071

[13] Kim SH Kamaya A Willmann JK. CT Perfusion of the Liver: Principles and Applications in Oncology. Radiology (2014) 272(2):322–44. doi: 10.1148/radiol.14130091 PMC4263626 25058132

[14] Yu Y Wang X Cen S Su H Hu C. Spectral Computed Tomography Imaging in the Differential Diagnosis of Lung Cancer and Inflammatory Myofibroblastic Tumor. J Comput Assist Tomography (2019) 43(2):1. doi: 10.1097/RCT.0000000000000840 30762653

[15] Yuan X Jing Z Quan C Cao J Ao G Tian Y. Differentiation of Malignant and Benign Pulmonary Nodules With First-Pass Dual-Input Perfusion CT. Eur Radiol (2013) 23(9):2469–74. doi: 10.1007/s00330-013-2842-x 23793548

[16] ohlsen DB Talakic E Fritz GA Quehenberger F Tillich M Schoellnast H. First Pass Dual Input Volume CT-Perfusion of Lung Lesions: The Influence of the CT- Value Range Settings on the Perfusion Values of Benign and Malignant Entities. Eur J Radiol (2016) 85(6):1109–14. doi: 10.1016/j.ejrad.2016.03.013 27161059

[17] Zhang G Cao Y Zhang J Zhao Z Zhang W Huang L. Focal Organizing Pneumonia in Patients: Differentiation From Solitary Bronchioloalveolar Carcinoma Using Dual-Energy Spectral Computed Tomography. Am J Trans Res (2020) 12(7):3974–83. PMC7407697 32774750

[18] Zhang Z Zou H Yuan A Jiang F Gong L. A Single Enhanced Dual-Energy CT Scan May Distinguish Lung Squamous Cell Carcinoma From Adenocarcinoma During the Venous Phase. Acad Radiol (2019) 27(5):624–9. doi: 10.1016/j.acra.2019.07.018 31447258

[19] Chen ML Li XT Wei YY Qi LP Sun YS. Can Spectral Computed Tomography Imaging Improve the Differentiation Between Malignant and Benign Pulmonary Lesions Manifesting as Solitary Pure Ground Glass, Mixed Ground Glass, and Solid Nodules? Thorac Cancer (2019) 10(2):234–42. doi: 10.1111/1759-7714.12937 PMC6360238 30582292

[20] Hou H Xu Z Zhang H Yan X. Combination Diagnosis of Multi-Slice Spiral Computed Tomography and Secretary Phospholipase A2-IIa for Solitary Pulmonary Nodules. J Clin Lab Anal (2017) 32(2):e22250. doi: 10.1002/jcla.22250 PMC6817176 28493533

[21] Yu L Leng S Mccollough CH. Dual-Energy CT–Based Monochromatic Imaging. Ajr Am J Roentgenol (2012) 199(5_supplement):S9. doi: 10.2214/AJR.12.9121 23097173

[22] Aaron S Savvas N. Spectral Computed Tomography: Fundamental Principles and Recent Developments. Korean J Radiol (2020) 1):86–96. doi: 10.3348/kjr.2020.0144 PMC7772378 32932564

[23] Sun Y Yang M Mao D Lv F Yin Y Li M. Low-Dose Volume Perfusion Computed Tomography (VPCT) for Diagnosis of Solitary Pulmonary Nodules. Eur J Radiol (2016) 85(6):1208–18. doi: 10.1016/j.ejrad.2016.03.026 27161072

[24] Xiong AZ Liu AJK Hu BCP Zhou AH Zhou AML Chen AW. Role of Immature Microvessels in Assessing the Relationship Between CT Perfusion Characteristics and Differentiation Grade in Lung Cancer. Arch Med Res (2010) 41(8):611–7. doi: 10.1016/j.arcmed.2010.11.005 21199730

[25] Yabuuchi H Kawanami S Iwama E Okamoto I Kamitani T Sagiyama K. Prediction of Therapeutic Effect of Chemotherapy for NSCLC Using Dual-Input Perfusion CT Analysis: Comparison Among Bevacizumab Treatment, Two-Agent Platinum-Based Therapy Without Bevacizumab, and Other Non-Bevacizumab Treatment Groups. Radiology (2018) 286(2):162204. doi: 10.1148/radiol.2017162204 29059037

[26] Zussman BM Boghosian G Gorniak RJ Olszewski ME Flanders AE. The Relative Effect of Vendor Variability in CT Perfusion Results: A Method Comparison Study. Ajr Am J Roentgenol (2011) 197(2):468–73. doi: 10.2214/AJR.10.6058 21785096

[27] Kudo K Sasaki M Yamada K Momoshima S Utsunomiya H Shirato H. Differences in CT Perfusion Maps Generated by Different Commercial Software: Quantitative Analysis by Using Identical Source Data of Acute Stroke Patients. Radiology (2010) 254(1):200–9. doi: 10.1148/radiol.254082000 20032153

[28] Chen X Xu Y Duan J Li C Sun H Wang W. Correlation of Iodine Uptake and Perfusion Parameters Between Dual-Energy CT Imaging and First-Pass Dual-Input Perfusion CT in Lung Cancer. Medicine (2017) 96(28):e7479. doi: 10.1097/MD.0000000000007479 28700488 PMC5515760

[29] Gordic S Puippe GD Krauss B Klotz E Desbiolles L Lesurtel M. Correlation Between Dual-Energy and Perfusion CT in Patients With Hepatocellular Carcinoma. Radiology (2016) 280(1):78–87. doi: 10.1148/radiol.2015151560 26824712

[30] Bao J Liu A Zhao C Hao F Su X Bao L. Correlation Between Dual-Energy Computed Tomography Single Scan and Computed Tomography Perfusion for Pancreatic Cancer Patients: Initial Experience. J Comput Assist Tomography (2019) 43(4):599–604. doi: 10.1097/RCT.0000000000000878 31162238

[31] Neri E Gabelloni M Buerle T Beets-Tan R Laghi A. Involvement of Radiologists in Oncologic Multidisciplinary Team Meetings: An International Survey by the European Society of Oncologic Imaging. Eur Radiol (2020) 31(2):983–91. doi: 10.1007/s00330-020-07178-w PMC7813742 32833089

[32] Liu J Chai Y Zhou J Dong C Zhang W Liu B. Spectral Computed Tomography Imaging of Gastric Schwannoma and Gastric Stromal Tumor. J Comput Assist Tomography (2017) 41(3):417–21. doi: 10.1097/RCT.0000000000000548 28505624

[33] Zhao J Chai Y Zhou J Zhang Z Wang Z. Energy Spectrum Computed Tomography Improves the Differentiation Between Benign and Malignant Solitary Pulmonary Nodules. Clin Invest Med Med Clinique Experimentale (2019) 42(3):E40–6. doi: 10.25011/cim.v42i3.33091 31563159

[34] Wang G Zhang C Deng K. Preliminary Application of High-Definition Computed Tomographic Gemstone Spectral Imaging in Lung Cancer. J Comput Assist Tomography (2014) 38(1):77–81. doi: 10.1097/RCT.0b013e3182a21633 24378884

[35] Tang H Yu N Jia Y Yu Y Duan H Han D. Assessment of Noise Reduction Potential and Image Quality Improvement of a New Generation Adaptive Statistical Iterative Reconstruction (ASIR-V) in Chest Computed Tomography. Br J Radiol (2018) 91(1081):20170521. doi: 10.1259/bjr.20170521 29076347 PMC5966217

